# Pilot study on CHCF1 genotype in a pig challenge model for enterotoxigenic *Escherichia coli* F4ab/ac associated post-weaning diarrhea

**DOI:** 10.1186/s12917-022-03474-3

**Published:** 2022-11-01

**Authors:** Martin Peter Rydal, Michela Gambino, Claus Bøttcher Jørgensen, Louise Ladefoged Poulsen, Lone Brøndsted, Jens Peter Nielsen

**Affiliations:** Department of Veterinary and Animal Sciences, Faculty of Health and Medical Sciences, University of Copenhagen, 1870 Frederiksberg C, Denmark

**Keywords:** Diarrhea, Inoculum dose, Experimental infection, Enterotoxigenic *Escherichia coli*, F4 fimbriae, Genetic marker, Piglet, Post-weaning

## Abstract

Host genotype is important for enterotoxigenic *E. coli* (ETEC) susceptibility. We conducted two trials to evaluate the effect of CHCF1 genotype on incidence of ETEC diarrhea. In trial 1 (*n* = 15 pigs), pigs were inoculated with 10^8^ CFU or 10^10^ CFU doses of an ETEC F4ac strain. In trial 2 (*n* = 33 pigs), pigs were inoculated with ETEC F4ab or F4ac. Across trials, all inoculated pigs that developed ETEC diarrhea were CHCF1 heterozygous susceptible (6/6). No inoculated CHCF1 homozygous resistant pigs developed ETEC diarrhea (0/26). Susceptibility towards ETEC F4ac/ab infection might correspond with CHCF1 genotype.

**Supplementary Information:**

The online version contains supplementary material available at 10.1186/s12917-022-03474-3.

## Introduction

Post-weaning diarrhea (PWD) is a widespread condition in pigs that can lead to morbidity and even death in the first weeks after weaning. Enterotoxigenic *E. coli* (ETEC) is the most common pathogen isolated from PWD outbreaks [1] and is characterized by fimbriae and enterotoxin production [2]. The fimbriae of ETEC adhere to specific host receptors and thereby enable colonization of the piglet intestine [2]. ETEC isolates from cases of PWD usually carry genes for F4 or F18 fimbriae [3, 4] that can be further subtyped according to their antigenic variant: F4ab, F4ac, and F4ad and F18ab and F18ac [5, 6]. The heat stable (ST) and heat labile enterotoxins (LT) produced by ETEC affect the electrolyte balance of the enterocytes and cause secretory diarrhea [2].

A locus responsible for ETEC F4ab/ac susceptibility has been mapped to pig chromosome 13 [7]. An intronic single nucleotide polymorphism (SNP) in the MUC4 gene [8] is often used to genotype for ETEC F4ab/ac susceptibility in porcine challenge studies [9–11]. However, association between the MUC4 genotype and susceptibility to ETEC F4ab/ac challenge is not perfect [12]. Recently, CHCF1 was indicated as a superior genetic marker to MUC4 for predicting F4ac receptor expression [13]. Further investigation into CHCF1 as a marker for ETEC F4ab/ac susceptibility in vivo is needed. In this study, we evaluated the marker in pigs experimentally infected with ETEC F4.

## Methods

### Animals

The study consisted of two trials with a total of 48 female pigs, Duroc x Landrace x Yorkshire, with no history of disease, acquired from a large Danish pig production herd. Pigs were weaned at PND 22 in trial 1 (*n* = 15) and PND 23 in trial 2 (*n* = 33). Experimental groups were housed in separate rooms with one pen per room. Wood shavings and straw was used as bedding on concrete floors. Rooms were kept at 26 ^o^ C and piglets had access to a 30–32 ^o^ C heated resting area. Animals were fed ad libitum with a standard weaner diet without added antimicrobials, Additional file 1.

## Experimental design

At weaning, the three most middle weight pigs of each of five (trial 1) and eleven (trial 2) litters were randomized into experimental groups to balance the study on sow level and reduce weight variation. In trial 1, the three groups of five pigs each were; 1) ET10 (F4ac, STb, LT) 10^8^ CFU, 2) ET10 10^10^ CFU, and 3) saline control. In trial 2, the three groups of 11 pigs each were; 1) ET10 10^10^ CFU, 2) ET54 (F4ab, STb, LT) 10^10^ CFU, and 3) saline control. Pigs were acclimatized for 1 day and inoculated once daily at day 2–3 (trial 1) and day 2–6 (trial 2). For a total of two inoculations in trial 1 and five in trial 2. The study period was 6 days post initial inoculation. At the end of the study period, pigs were sedated with intramuscular injection of 1 mL/10 kg zoletil mix (ZOLETIL® 50 VET, Virbac, Kolding, Denmark (25 mg/mL tiletamine hydrochloride and 25 mg/mL zolazepam hydrochloride) (1 bottle) + 1.25 ml ketamine (100 mg/mL) + 6.25 mL xylazine (20 mg/mL) + 2.5 mL butorphanol (10 mg/mL)) and euthanized with pentobarbital by intracardiac injection. The experimental unit was the pig. Protocols were registered at the Department of Experimental Medicine, University of Copenhagen. The investigators were blinded towards genotypes during the conduct of the study.

## Inoculum

ET10 (O149:H10, F4ac, STb, LT) was isolated from a mild clinical case of PWD, whereas ET54 (O149:H10, F4ab, STb, LT) was isolated from a severe clinical case of PWD. Whole genome sequences of ET10 and ET54 are available at NCBI (BioProject ID: PRJNA770188). Briefly, 1 L bacterial inocula in LB broth (BD Difco LB broth, Lennox, BD 240,230, Fisher Scientific, USA) were centrifuged, washed and resuspended in sterile saline. While in trial 1, ET10 was harvested in stationary phase, in trial 2 ET54 and ET10 were harvested in late exponential phase. Size 1 gelatine capsules (CapsulCN, China) were filled with 0.4 mL of saline or the bacterial suspensions, snap frozen in dry ice and stored at −20 °C up to a week before inoculating pigs. The bacterial count from the capsules was verified by spotting on blood agar plates (BA, 5% calf blood in blood agar base, ThermoFischer, CM0055) regularly until inoculation. In trial 1, at each inoculation, pigs received two capsules of 1 × 10^8^ CFU/0.4 mL in the low dose group or one capsule of 5 × 10^10^ CFU/0.4 mL in the high dose group. In trial 2, at each inoculation, pigs received one capsule of 2 × 10^10^ CFU/0.4 mL of ET10 or ET54.

## Clinical examination

In trial 1, fecal samples were collected morning and evening and scored visually 1 to 4 based on consistency [14]. Diarrhea was defined as fecal score ≥ 3 and ETEC diarrhea was defined as diarrhea and simultaneous isolation of ETEC from the diarrhea sample. Fecal dry matter percentage was calculated by weighing feces before and after drying to constant weight, in an oven at 75 °C for 18 h. The procedure was the same in trial 2 except that fecal samples were collected only in the morning. Pigs were monitored at least twice daily and scored according to Additional file 2.

## Microbiological analysis

Rectal swabs were collected from pigs in the herd for ETEC diagnosis at weaning. In trial 1, rectal swabs for microbiology were collected from each animal twice daily, streaked on BA and incubated at 37 °C ON. Shedding of haemolytic *E. coli* was assessed based on presence in primary, secondary or tertiary streak. One haemolytic *E. coli* colony per pig per day was subcultured on BA, the virulence factors were identified by PCR (F4, F18 as in [15]) and typed and compared with the challenge strain by Pulsed-field Gel Electrophoresis (PFGE) [16]. In trial 2, rectal swabs were collected once daily and daily shedding of haemolytic *E. coli* was instead evaluated semi-quantitatively as percentage haemolytic *E. coli* out of total bacterial growth (0–100%). Multiplex PCR [15] was used to identify virulence factors (F4, F18 and STb, STa, LT) of one haemolytic isolate per pig per day and PFGE was used to compare the challenge strain with a subset of 52 samples across groups.

## DNA-marker based tests

Genomic DNA was extracted from 200 µl EDTA-stabilized blood from each pig using the MasterPure™ DNA purification Kit (Epicentre, Madison, Wisconsin, USA). TaqMan SNP Genotyping assays were designed by Thermo Fisher Scientific (Waltham, Massachusetts, USA).

A total of 25 ng genomic DNA was used for genotyping each of the three markers by TaqMan according to the manufacturer’s instructions. Allele calling was performed on a Mx3000P qPCR System (Agilent, Santa Clara, California, USA).

## Statistics

The primary outcome was ETEC diarrhea. Secondary outcomes were: diarrhea, fecal dry matter content, haemolytic *E. coli* shedding, days of challenge strain shedding, and body weight gain.

Data analysis was performed with R version 4.1.1 [17] and visualized with Graphpad Prism 9 (GraphPad Software, San Diego, CA, USA). *p*-values < 0.05 were considered significant. Data was assessed for normal distribution using Q-Q-plots.

Proportion of animals with ETEC diarrhea and diarrhea were analyzed using Firth’s logistic regression [18]. Days of challenge strain shedding, fecal dry matter differences between fecal scores, and weight gain were analyzed with Kruskal Wallis. Daily shedding of haemolytic *E. coli* and daily fecal dry matter was analyzed with Kruskal Wallis. FDR adjustment was applied on raw *p*-values to correct for multiple comparisons. Post hoc, Dunn’s test [19] was used.

## Results

### Animals for the study

At the farm, culturing of rectal swabs from enrolled pigs revealed no ETEC and no diarrhea was detected (data not shown). At arrival at the experimental facility and before inoculation, pigs were still healthy and had no perianal fecal staining or diarrhea. Weaning weight ranged from 3.8 to 8.9 kg. Mean birthweight and weaning weight were similar between groups and trials, Additional file 3.

## Progression of ETEC diarrhea

Fecal consistency of ETEC diarrhea varied between loose (score 3) and watery (score 4). In trial 2, we observed single events of diarrhea in four pigs outside the designated sampling in connection with the fourth and fifth inoculation procedure. These diarrhea recordings were seemingly stress-induced as no diarrhea was found subsequently and the recordings were therefore excluded from the study.

In trial 1, 1 out of 5 pigs developed ETEC diarrhea in both the 10^8^ CFU and 10^10^ CFU group. All ETEC isolates from diarrheal fecal samples had F4 fimbriae encoding genes and were confirmed to be the challenge strain by profile comparison with PFGE.

In trial 2, 2 out of 11 pigs developed ETEC diarrhea in both the ET10 and ET54 group. All ETEC isolated from diarrhea samples had virotype F4, STb, LT and PFGE confirmed that the strain ET54 was only found in the ET54 group and that the strain ET10 was only found in the ET10 group.

## Shedding of haemolytic *E. coli* and challenge strain

In trial 1, shedding of haemolytic *E. coli* occurred in most pigs, across all groups, at some point during the study period. At baseline, only one pig shed haemolytic *E. coli*, but no genes for F4 or F18 fimbriae were detected in this isolate. All isolates that were positive for F4 were confirmed to be the challenge strain by PFGE. No genes for F4 fimbriae were detected in *E. coli* isolated from any piglets in the saline control group, confirming the absence of the challenge strain. At the end of the study, at 5 and 6 dpii, genes for F18 were detected in isolates from a single pig, but the shedding of F18 was not associated with diarrhea.

In trial 2, shedding of haemolytic *E. coli* also occurred for most pigs across all three groups. No genes encoding ETEC virulence factors were found in the *E. coli* isolated from the saline control group. Five pigs across the inoculation groups shed haemolytic *E. coli* at baseline, but none of these isolates encoded for ETEC virulence factors.

## Clinical condition

The diarrhea observed in the study was mild to severe lasting 1 to 3 days and was not associated with clinical signs of dehydration, fever, or hypothermia.

## Fecal dry matter content

Fecal dry matter was significantly different between fecal scores (χ2 = 131.54, df = 3, *p* < 0.0001), confirming the validity of the fecal score in assessing diarrhea in the study. The fecal dry matter percentages among fecal scores were as follows (data presented as means ± SD): Score 1 (*n* = 376, 30.74 ± 6.0%), score 2 (*n* = 38, 21.91 ± 4.3%), score 3 (*n* = 23, 12.8 ± 3.1%), score 4 (*n* = 8, 8.0 ± 2.1%). Five fecal samples were missing due to failure to collect sample/lost sample/dropped sample at random.

## Genotype effects

**Fig. 1:**
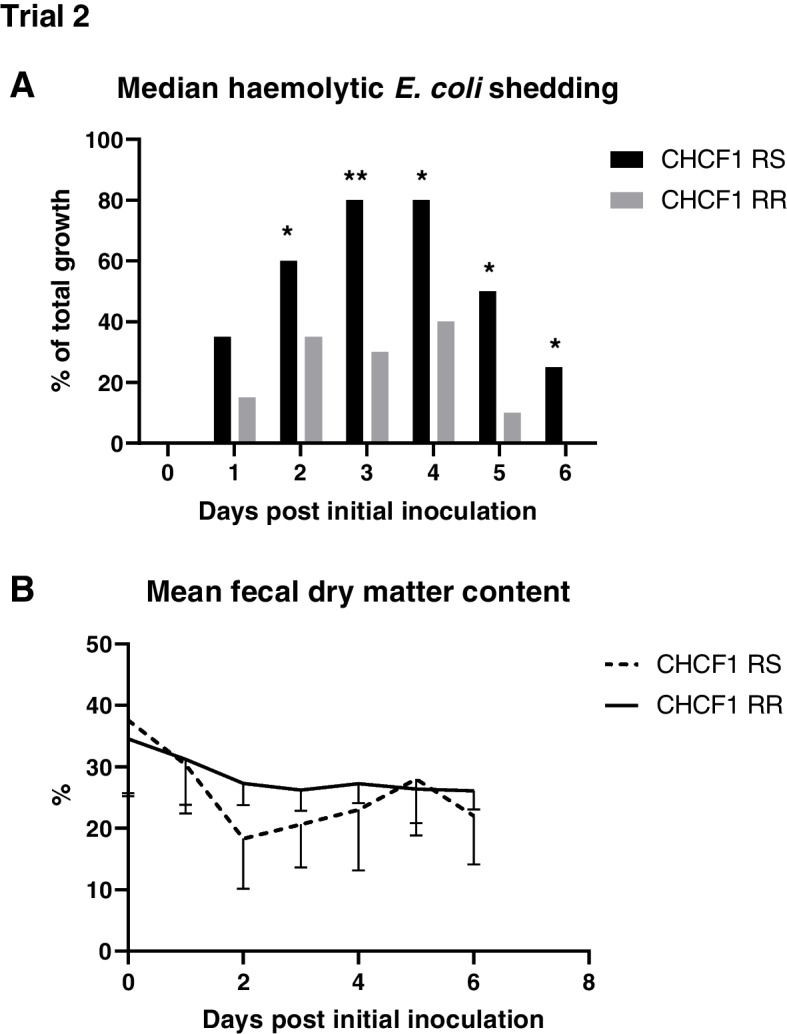
Haemolytic *E. coli* shedding and fecal dry matter after ETEC F4 challenge between CHCF1 genotypes. CHCF1 RR: *n* = 18, CHCF1 RS: *n* = 4 pigs. **A** In trial 2, shedding of haemolytic *E. coli* was assessed as percentage haemolytic *E. coli* out of total bacterial growth (0–100%). Data was analyzed for daily differences between groups with Kruskal Wallis, false discovery rate (FDR) adjustment was applied on *p*-values to correct for multiple comparisons, * *p* < 0.05, ** *p* < 0.01. **B** Data presented as means with standard deviation and analyzed for daily differences in fecal dry matter between groups with Kruskal–Wallis, FDR was applied to correct for multiple comparisons

According to FUT1, one pig was homozygous resistant (RR) against ETEC F18, 15 of 48 (31%) were heterozygous susceptible (RS), and the majority homozygous susceptible (SS) 32 of 48 (66%), Additional file 4. No spontaneous ETEC F18 diarrhea occurred. All pigs were genotyped as RR towards ETEC F4ab/ac according to the MUC4 marker. Six MUC4 resistant pigs (which were CHCF1 susceptible) developed ETEC F4 diarrhea. In contrast, CHCF1 genotyping indicated that 11 pigs were RS towards ETEC F4ab/ac. Out of these 11 RS pigs, six were located in challenge groups across trials. Comparison in primary and secondary outcomes between CHCF1 genotypes in challenge groups, across trials, are presented in Table 1. All six RS in challenge groups developed ETEC F4 diarrhea (6/6) whereas none with the RR profile in challenge groups developed ETEC F4 diarrhea. In challenge groups, pigs with CHCF1 RS genotype had challenge strain shedding for more days compared to CHCF1 RR, Table 1. Daily haemolytic *E. coli* shedding for CHCF1 genotype in the challenge groups of trial 2 are in Fig. 1A and Additional file 5. Significant differences in haemolytic *E. coli* shedding were found from 2 to 6 dpii. As for development in fecal dry matter content, numerically lower levels were seen in CHCF1 RS pigs, especially around 2 dpii, but no significant differences were found, Fig. 1B. In summary, all challenged pigs that were susceptible according to the CHCF1 marker developed ETEC diarrhea with affected secondary disease related outcomes, whereas CHCF1 resistant remained healthy after challenge with ETEC F4 ab/ac.

**Table 1:** Clinical and microbiological outcomes according to CHCF1 genotype: Combined results for trial 1 and 2

CHCF1 genotype	RS	RR	*p*-value
Pigs with ETEC diarrhea [n/n_group_]	6/6	0/26	< 0.0001
Pigs with diarrhea [n/n_group_]	6/6	4/26	0.0001
Mean weight gain (SD) [gram]	391 (356)	707 (464)	0.10
Mean challenge strain shedding (SD) [days]	4.8 (0.7)	1.3 (1.2)	0.0001

Data presented includes combined results from pigs of inoculation groups of trial 1 and 2. RS: heterozygous susceptible, *n* = 6 pigs. RR: homozygous resistant, *n* = 26 pigs. Diarrhea = fecal score ≥ 3. ETEC diarrhea = fecal score ≥ 3 with isolation of ETEC. Fecal score (firm and shaped (1), soft and shaped (2), loose (3), watery (4). *p*-values were generated using Kruskal–Wallis test for analysis of group differences in weight gain and days of challenge strain shedding. Difference in proportions of animals with diarrhea in study period analyzed with firth’s logistic regression. Significantly higher occurrence of ETEC diarrhea and diarrhea was found in RS pigs (coefficient (coef) = 6.53, 95% Confidence interval (CI) = 3.40;12.30 and coef = 4.17, 95% CI = 1.78;9.11, respectively) and more days of challenge strain shedding (χ2 = 13.95, df = 1)

## Discussion

In the study, we set out to test if the incidence and severity of ETEC diarrhea was affected by CHCF1 genotype, inoculum dose (10^8^ CFU versus 10^10^ CFU) and ETEC fimbriae subtype (F4ac versus F4ab). Interestingly, we found that only inoculated CHCF1 RS pigs developed ETEC diarrhea regardless of dose or strain, whereas CHCF1 RR pigs remained completely unaffected. The CHCF1 RS pigs also shed significantly more haemolytic *E. coli* and had more days of challenge strain shedding than the challenged CHCF1 RR pigs. As only a few CHCF1 RS pigs were found per challenge group we could not make inference on the effect of inoculation dose or fimbriae subtype on incidence or severity of ETEC diarrhea.

Genotyping with CHCF1 indicated that 11 out of 48 pigs across trials were RS towards ETEC F4ab/ac. All CHCF1 RS pigs in our challenge groups developed ETEC diarrhea, whereas no CHCF1 RR pigs developed ETEC diarrhea. Thus, suggesting a link between CHCF1 genotype and ETEC F4ab/ac susceptibility. In contrast, MUC4 genotyping indicated that all pigs were resistant and offered no agreement with diarrhea development. The agreement we found between CHCF1 genotype and ETEC diarrhea is stronger than what has been reported in literature for MUC4. For example, Jensen and colleagues [20] found 74% challenge strain associated diarrhea in MUC4 susceptible pigs, one day post inoculation. However, 40% of the challenged MUC4 resistant pigs in their study also developed challenge strain induced diarrhea a few days later. MUC4 resistant pigs that develop diarrhea after ETEC F4 challenge have been described recently [10]. We chose the MUC4 marker for comparison as it has been the most widely used genotype test for ETEC F4 susceptibility in challenge studies and is still being used [9, 10]. Other more recently proposed markers near the MUC13 gene that have been associated with high ETEC F4 susceptibility [21] could have been considered for comparison.

In conclusion, CHCF1 genotype was a better marker for F4ab/ac susceptibility in vivo than MUC4. This may contribute to explain why ETEC F4 strains continue to be isolated from PWD outbreaks in Denmark, despite the breeding programme (Danbred) where MUC4 RS genotypes were eliminated to obtain ETEC F4 resistance. In future challenge studies, CHCF1 genotyping to screen for susceptible pigs may be preferred to MUC4 to achieve a higher proportion of pigs with ETEC diarrhea. The present trial was not designed just to study the effect of genotype, but our observations suggest CHCF1 as a promising marker for screening experimental animals and thus it should be tested in a separate trial. Further studies are needed to elucidate if the same link we found between CHCF1 genotype and ETEC F4 susceptibility in vivo occur across herds and pig breeds.

## Supplementary Information

**Additional file 1: Additional file 1.** Feed formulation.

**Additional file 2: Additional file 2.** Clinical scoring of pig health.

**Additional file 3: Additional file 3.** Mean birthweight and weaning weight of pigs in trial 1 and trial 2. Description of data: ET10: ETEC F4ac, STb, LT. ET54: ETEC F4ab, STb, LT. Trial 1: Saline control (*n*=5 pigs), ET10, 10^8^ CFU (*n*=5 pigs), ET10, 10^10^ CFU (*n*=5 pigs). Trial 2: Saline control (*n*=11 pigs), ET10, 10^10^ CFU (*n*=11 pigs). ET54, 10^10^ CFU (*n*=11 pigs). Data presented as means ± standard deviation.

**Additional file 4: Additional file 4.** Distribution of genotypes for ETEC susceptibility according to MUC4, CHCF1 and FUT1. Description of data: RR: Homozygous resistant, RS: heterozygous susceptible, SS: homozygous susceptible. ET10: ETEC F4ac, STb,LT. Genotypes for ETEC F4ab/ac susceptibility were investigated according to the MUC4 and CHCF1 genotyping test. Genotypes for ETEC F18 susceptibility were investigated according to the FUT1 genotyping test.

**Additional file 5: Additional file 5.** Medians, interquartile ranges and analysis of haemolytic *E. coli* shedding between CHCF1 genotypes in challenge groups of trial 1 and trial 2 over time. Description of data: RR: homozygous resistant, RS: heterozygous susceptible. In trial 1, haemolytic *E. coli* shedding was assessed based on presence in primary/secondary/tertiary streak (score of 0: no growth, score 1: growth in primary streak, score 2: growth in secondary streak, score 3: growth in tertiary streak). In trial 2, shedding of haemolytic *E. coli* was assessed as percentage haemolytic *E. coli* out of total bacterial growth (0-100%). Data was analyzed for daily differences between groups with Kruskal Wallis, false discovery rate was applied on *p*-values to correct for multiple comparisons.

**Additional file 6: Additional file 6.** Arrive guidelines.

**Additional file 7: Additional file 7.** Datasets analyzed during the current study.

## Data Availability

All data generated or analysed during this study are included in this published article [and its supplementary information files (Additional file 7)]. SNPs can be found in the dbSNP data base: CHCF1 (dbSNP:rs340488770). MUC4 (dbSNP:rs338992994). FUT1 (dbSNP:rs335979375).

## References

[1] Rhouma M, Fairbrother JM, Beaudry F, Letellier A (2017). Post weaning diarrhea in pigs: risk factors and non-colistin-based control strategies. Acta Vet Scand.

[2] Dubreuil JD, Isaacson RE, Schifferli DM. Animal Enterotoxigenic Escherichia coli. EcoSal Plus. 2016. 10.1128/ecosalplus.ESP-0006-2016. 10.1128/ecosalplus.esp-0006-2016 PMC5123703 27735786

[3] Frydendahl K (2002). Prevalence of serogroups and virulence genes in Escherichia coli associated with postweaning diarrhoea and edema disease in pigs and a comparison of diagnostic approaches. Vet Microbiol.

[4] Luppi A, Gibellini M, Gin T, Vangroenweghe F, Vandenbroucke V, Bauerfeind R, Bonilauri P, Labarque G, Hidalgo Á (2016). Prevalence of virulence factors in enterotoxigenic Escherichia coli isolated from pigs with post-weaning diarrhoea in Europe. Porc Heal Manag.

[5] Guinee PAM, Jansen WH (1979). Behavior of Escherichia coli K antigens K88ab, K88ac, and K88ad in immunoelectrophoresis, double diffusion, and hemagglutination. Infect Immun.

[6] Rippinger P, Bertschinger HU, Imberechts H, Nagy B, Sorg I, Stamm M, Wild P, Wittig W (1995). Designations F18ab and F18ac for the related fimbrial types F107, 2134P and 8813 of Escherichia coli isolated from porcine postweaning diarrhoea and from oedema disease. Vet Microbiol.

[7] Edfors-Lilja I, Gustafsson U, Duval-Iflah Y, Ellergren H, Johansson M, Juneja RK, Marklund L, Andersson L (1995). The porcine intestinal receptor for Escherichia coli K88ab, K88ac: regional localization on chromosome 13 and influence of IgG response to the K88 antigen. Anim Genet.

[8] Jørgensen CB, Cirera S, Archibald AL, Andersson L, Fredholm M, Edfors-Lilja I. Porcine polymorphisms and methods for detecting them. 2004. International patent number: WO/2004/ 048606.

[9] Sterndale SO, Miller DW, Mansfield JP, Kim JC, O’Dea M, Pluske JR (2019). Technical note: novel delivery methods for an enterotoxigenic Escherichia coli infection model in MUC4-locus sequenced weaner pigs. J Anim Sci.

[10] Matsumoto H, Miyagawa M, Takahashi S, Shima R, Oosumi T (2020). Improvement of the Enterotoxigenic Escherichia coli Infection Model for Post-Weaning Diarrhea by Controlling for Bacterial Adhesion, Pig Breed and MUC4 Genotype. Vet Sci.

[11] van Hees H, Maes D, Millet S, den Hartog L, van Kempen T, Janssens G. Fibre supplementation to pre-weaning piglet diets did not improve the resilience towards a post-weaning enterotoxigenic E. coli challenge. J Anim Physiol Anim Nutr (Berl.) 2020. 10.1111/jpn.13475. 10.1111/jpn.13475 33241907

[12] Rasschaert K, Verdonck F, Goddeeris BM, Duchateau L, Cox E. Screening of pigs resistant to F4 enterotoxigenic Escherichia coli (ETEC) infection. Vet Microbiol. 2007. 10.1016/J.VETMIC.2007.02.017. 10.1016/j.vetmic.2007.02.017 17399917

[13] Hu D, Rampoldi A, Bratus-Neuenschwander A, Hofer A, Bertschinger HU, Vögeli P, Neuenschwander S (2019). Effective genetic markers for identifying the Escherichia coli F4ac receptor status of pigs. Anim Genet.

[14] Pedersen KS, Toft N (2011). Intra- and inter-observer agreement when using a descriptive classification scale for clinical assessment of faecal consistency in growing pigs. Prev Vet Med.

[15] Zhang W, Zhao M, Ruesch L, Omot A, Francis D (2007). Prevalence of virulence genes in Escherichia coli strains recently isolated from young pigs with diarrhea in the US. Vet Microbiol.

[16] PulseNet, C.D.C.. Standard Operating Procedure for PulseNet PFGE of Escherichia coli O157:H7, Escherichia coli non-O157 (STEC), Salmonella serotypes, Shigella sonnei and Shigella flexneri. 2017. URL https://www.cdc.gov/pulsenet/pdf/ecoli-shigella-salmonella-pfge-protocol-508c.pdf. Accessed 13 Jan 2022.

[17] R Core Team. R: A language and environment for statistical computing. Vienna: R Foundation for Statistical Computing; 2021. URL https://www.R-project.org/. Accessed 13 Jan 2022.

[18] Heinze G, Ploner M, Jiricka L. logistf: Firth's Bias-Reduced Logistic Regression. R package version 1.24; 2020. URL https://cran.r-project.org/web/packages/logistf/index.html. Accessed 13 Jan 2022.

[19] Ogle DH, Doll JC, Wheeler P, Dinno A. FSA: Fisheries Stock Analysis. R package version 0.9.1; 2021. URL https://github.com/droglenc/FSA. Accessed 13 Jan 2022.

[20] Jensen GM, Frydendahl K, Svendsen O, Jørgensen CB, Cirera S, Fredholm M, Nielsen J-P, Møller K (2006). Experimental infection with Escherichia coli O149:F4ac in weaned piglets. Vet Microbiol.

[21] Goetstouwers T, Van Poucke M, Coppieters W, Nguyen VU, Melkebeek V, Coddens A, Van Steendam K, Deforce D, Cox E, Peelman LJ. Refined candidate region for F4ab/ac enterotoxigenic *Escherichia coli* susceptibility situated proximal to *MUC13* in pigs. PLoS One. 2014. 10.1371/journal.pone.0105013 10.1371/journal.pone.0105013 PMC4138166 25137053

