# Supplementary material for: Pilot study on CHCF1 genotype in a pig challenge model for enterotoxigenic Escherichia coli F4ab/ac associated post-weaning diarrhea
Source: BMC Vet Res. 2022 Nov 1;18:382. doi: 10.1186/s12917-022-03474-3 (PMC9624054; doi:10.1186/s12917-022-03474-3)
Supplement: Supplementary file 2 — Additional file 2: Additional file 2. Clinical scoring of pig health. [file 12917_2022_3474_MOESM2_ESM.docx]

**Clinical examination**

Clinical examination included daily assessment of wet perianal fecal staining, clinical signs of dehydration (skin turgor with skin pinch test, sunken eyes and flanks, rough hair coat) rectal temperature and body weight.

**1 = Healthy pig, no symptoms of disease**

Bright, Alert and Responsive (BAR), pink skin color, rhythmic thoraco-abdominal breathing (respiration rate 20-50), extremities are warm, normal feed and water intake, normal gait, normal temperature (39ºC. Lower critical point: 38.4 ºC. Upper critical point: 40 ºC).

**2 = Mild symptoms of disease/discomfort, supportive therapy may be initiated**

BAR/or Quite, Alert and Responsive, increased respiration rate (>50), reduced appetite and reduced activity, mild signs of inflammation (e.g. around perianal area), mild lameness, mild GI-symptoms (e.g. diarrhea/vomiting, perianal staining), moderate dehydration (mildly sunken eyes, rough sticky wet haircoat), fever (40ºC<).

**3 = Moderate/severe symptoms of disease, treatment initiated/euthanasia considered**

Lethargic, bloody diarrhea, respiratory distress, whitish/pale conjunctiva, lying down most of the time, little/or no food and water consumption, perianal staining, severe dehydration (sunken eyes, rough sticky wet haircoat, weight loss, cachexia), moderate/severe lameness, moderate/severe GI-symptoms (frequent watery diarrhea, frequent vomiting), fever (40ºC<).

If an animal received a score 3 (did not occur in the study), appropriate treatment would be initiated, if clinical signs would not improve within the next 3 hours the pig would be euthanized.
