# Supplementary material for: Pilot study on CHCF1 genotype in a pig challenge model for enterotoxigenic Escherichia coli F4ab/ac associated post-weaning diarrhea
Source: BMC Vet Res. 2022 Nov 1;18:382. doi: 10.1186/s12917-022-03474-3 (PMC9624054; doi:10.1186/s12917-022-03474-3)
Supplement: Supplementary file 3 — Additional file 3: Additional file 3. Mean birthweight and weaning weight of pigs in trial 1 and trial 2. Description of data: ET10: ETEC F4ac, STb, LT. ET54: ETEC F4ab, STb, LT. Trial 1: Saline control (n=5 pigs), ET10, 108 CFU (n=5 pigs), ET10, 1010 CFU (n=5 pigs). Trial 2: Saline control (n=11 pigs), ET10, 1010 CFU (n=11 pigs). ET54, 1010 CFU (n=11 pigs). Data presented as means ± standard deviation. [file 12917_2022_3474_MOESM3_ESM.docx]

| **Additional file 3**. Mean birthweight and weaning weight of pigs in trial 1 and trial 2 | | | | | | | |
| --- | --- | --- | --- | --- | --- | --- | --- |
|  | Trial 1 | | |  | Trial 2 | | |
| Groups | Saline  control | ET10,  10^8^ CFU | ET10,  10^10^ CFU |  | Saline  control | ET10,  10^10^ CFU | ET54,  10^10^ CFU |
| Birthweight [kg] | 1.4 (0.3) | 1.5 (0.2) | 1.5 (0.2) |  | 1.4 (0.1) | 1.2 (0.2) | 1.3 (0.2) |
| Weaning weight [kg] | 6.4 (1.5) | 6.7 (1.7) | 6.5 (1.4) |  | 6.0 (0.9) | 6.0 (1.2) | 6.0 (1.0) |
