# Supplementary material for: Pilot study on CHCF1 genotype in a pig challenge model for enterotoxigenic Escherichia coli F4ab/ac associated post-weaning diarrhea
Source: BMC Vet Res. 2022 Nov 1;18:382. doi: 10.1186/s12917-022-03474-3 (PMC9624054; doi:10.1186/s12917-022-03474-3)
Supplement: Supplementary file 4 — Additional file 4: Additional file 4. Distribution of genotypes for ETEC susceptibility according to MUC4, CHCF1 and FUT1. Description of data: RR: Homozygous resistant, RS: heterozygous susceptible, SS: homozygous susceptible. ET10: ETEC F4ac, STb,LT. Genotypes for ETEC F4ab/ac susceptibility were investigated according to the MUC4 and CHCF1 genotyping test. Genotypes for ETEC F18 susceptibility were investigated according to the FUT1 genotyping test. [file 12917_2022_3474_MOESM4_ESM.docx]

| **Additional file 4. Distribution of genotypes for ETEC susceptibility according to MUC4, CHCF1 and FUT1** | | | | | | | | | | | | | | | | |
| --- | --- | --- | --- | --- | --- | --- | --- | --- | --- | --- | --- | --- | --- | --- | --- | --- |
| **Trial 1** | |  | | | | | |  | | | | |  | | | |
| Groups | | Saline control | | | | | | ET10, 10^8^ CFU | | | | | ET10, 10^10^ CFU | | | |
| Genotype profile | | RR | | RS | | SS | | RR | | RS | SS | | RR | RS | SS |  |
| MUC4 | | 5 | | 0 | | 0 | | 5 | | 0 | 0 | | 5 | 0 | 0 |  |
| CHCF1 | | 1 | | 4 | | 0 | | 4 | | 1 | 0 | | 4 | 1 | 0 |  |
| FUT1 | | 0 | | 2 | | 3 | | 0 | | 2 | 3 | | 0 | 2 | 3 |  |
| **Trial 2** |  | | | | | |  | | | | |  | | | | |
| Groups | Saline control | | | | | | ET10, 10^10^ CFU | | | | | ET54, 10^10^ CFU | | | | |
| Genotype profile | RR | | RS | | SS | | RR | | RS | | SS | RR | | RS | SS | |
| MUC 4 | 11 | | 0 | | 0 | | 11 | | 0 | | 0 | 11 | | 0 | 0 | |
| CHCF1 | 10 | | 1 | | 0 | | 9 | | 2 | | 0 | 9 | | 2 | 0 | |
| FUT1 | 0 | | 4 | | 7 | | 1 | | 1 | | 9 | 0 | | 4 | 7 | |
