# Supplementary material for: Pilot study on CHCF1 genotype in a pig challenge model for enterotoxigenic Escherichia coli F4ab/ac associated post-weaning diarrhea
Source: BMC Vet Res. 2022 Nov 1;18:382. doi: 10.1186/s12917-022-03474-3 (PMC9624054; doi:10.1186/s12917-022-03474-3)
Supplement: Supplementary file 5 — Additional file 5: Additional file 5. Medians, interquartile ranges and analysis of haemolytic E. coli shedding between CHCF1 genotypes in challenge groups of trial 1 and trial 2 over time. Description of data: RR: homozygous resistant, RS: heterozygous susceptible. In trial 1, haemolytic E. coli shedding was assessed based on presence in primary/secondary/tertiary streak (score of 0: no growth, score 1: growth in primary streak, score 2: growth in secondary streak, score 3: growth in tertiary streak). In trial 2, shedding of haemolytic E. coli was assessed as percentage haemolytic E. coli out of total bacterial growth (0-100%). Data was analyzed for daily differences between groups with Kruskal Wallis, false discovery rate was applied on p-values to correct for multiple comparisons. [file 12917_2022_3474_MOESM5_ESM.docx]

| **Additional file 5**. Medians, interquartile ranges and analysis of hemolytic *E. coli* shedding between CHCF1 genotypes in challenge groups of trial 1 and trial 2 over time | | | | | | | | | | | | |
| --- | --- | --- | --- | --- | --- | --- | --- | --- | --- | --- | --- | --- |
|  | Trial 1 | | | | | | | | | | |  |
|  | CHCF1 RR (n=8 pigs) | | | | |  | CHCF1 RS (n=2 pigs) | | | | |  |
|  | Min | Q1 | Median | Q3 | Max |  | Min | Q1 | Median | Q3 | Max | p.adj-value |
| Day 0 | 0 | 0 | 0 | 0 | 1 |  | 0 | 0 | 0 | 0 | 0 | 0.65 |
| Day 0.5 | 0 | 0 | 0 | 0 | 0 |  | 0 | 0 | 0 | 0 | 0 | - |
| Day 1 | 0 | 0 | 0.5 | 1 | 3 |  | 1 | 1.5 | 2 | 2.5 | 3 | 0.25 |
| Day 1.5 | 0 | 0 | 0 | 0.5 | 2 |  | 0 | 0.75 | 1.5 | 2.25 | 3 | 0.43 |
| Day 2 | 0 | 0 | 1 | 1.5 | 3 |  | 2 | 2.25 | 2.5 | 2.75 | 3 | 0.25 |
| Day 2.5 | 0 | 0.75 | 1 | 2 | 3 |  | 3 | 3 | 3 | 3 | 3 | 0.15 |
| Day 3 | 0 | 0 | 0 | 0 | 2 |  | 3 | 3 | 3 | 3 | 3 | 0.1 |
| Day 3.5 | 0 | 0 | 0 | 0.25 | 2 |  | 3 | 3 | 3 | 3 | 3 | 0.1 |
| Day 4 | 0 | 0 | 0 | 1 | 2 |  | 3 | 3 | 3 | 3 | 3 | 0.1 |
| Day 4.5 | 0 | 0 | 1 | 2.5 | 3 |  | 3 | 3 | 3 | 3 | 3 | 0.22 |
| Day 5 | 0 | 0 | 0 | 1.5 | 3 |  | 0 | 0.75 | 1.5 | 2.25 | 3 | 0.65 |
| Day 5.5 | 0 | 0 | 0 | 2 | 3 |  | 2 | 2.25 | 2.5 | 2.5 | 3 | 0.22 |
| Day 6 | 0 | 0 | 0 | 0.75 | 3 |  | 0 | 0.75 | 1.5 | 2.25 | 3 | 0.6 |
| Day 6.5 | 0 | 0 | 0 | 2 | 3 |  | 3 | 3 | 3 | 3 | 3 | 0.15 |
|  |  |  |  |  |  |  |  |  |  |  |  |  |
|  | Trial 2 | | | | | | | | | | |  |
|  | CHCF1 RR (n=18 pigs) | | | | |  | CHCF1 RS (n=4 pigs) | | | | |  |
|  | Min | Q1 | Median | Q3 | Max |  | Min | Q1 | Median | Q3 | Max | p.adj-value |
| Day 0 | 0 | 0 | 0 | 37.5 | 60 |  | 0 | 0 | 0 | 0 | 0 | 0.24 |
| Day 1 | 0 | 0 | 15 | 30 | 40 |  | 0 | 22.5 | 35 | 42.5 | 50 | 0.21 |
| Day 2 | 0 | 20 | 35 | 40 | 60 |  | 40 | 55 | 60 | 62.5 | 70 | 0.02 |
| Day 3 | 5 | 30 | 30 | 40 | 90 |  | 70 | 77.5 | 80 | 83.75 | 95 | 0.003 |
| Day 4 | 0 | 10 | 40 | 60 | 90 |  | 70 | 80 | 80 | 80 | 80 | 0.01 |
| Day 5 | 0 | 2.5 | 10 | 20 | 90 |  | 20 | 27.5 | 50 | 72.5 | 80 | 0.04 |
| Day 6 | 0 | 0 | 0 | 3.75 | 10 |  | 0 | 15 | 25 | 30 | 30 | 0.02 |
